# Comparative Transcriptome Analysis of Cell-Free Fetal RNA from Amniotic Fluid and RNA from Amniocytes in Uncomplicated Pregnancies

**DOI:** 10.1371/journal.pone.0132955

**Published:** 2015-07-16

**Authors:** J. H. Kang, H. J. Park, Y. W. Jung, S. H. Shim, S. R. Sung, J. E. Park, D. H. Cha, E. H Ahn

**Affiliations:** 1 Department of Obstetrics and Gynecology, CHA Graduate School of Medicine, CHA University, Seoul, Republic of Korea; 2 Department of Obstetrics and Gynecology, CHA Gangnam Medical Center, CHA University, Seoul, Republic of Korea; 3 Genetic Laboratory, Fertility Center of CHA Gangnam Medical Center, CHA University, Seoul, Republic of Korea; 4 Department of Obstetrics and Gynecology, CHA Bundang Medical Center, CHA University, Seongnam, Republic of Korea; 5 Department of Obstetrics and Gynecology, Zion Women’s hospital, Suwon, Republic of Korea; University of Bonn, Institut of experimental hematology and transfusion medicine, GERMANY

## Abstract

**Objectives:**

We aimed to compare tissue-specific expression profiles and biological pathways of RNA from amniocytes and amniotic fluid supernatant (AFS) from second-trimester pregnancies by using transcriptome analysis. Additionally, we wanted to explore whether cell-free RNA from AFS exhibits a unique gene expression signature that more adequately reflects the fetal developmental process than amniocyte RNA.

**Methods:**

Amniotic fluid samples were prospectively collected in the second trimester of pregnancy from euploid fetuses. Total RNA was extracted from amniocytes and AFS and hybridized to Affymetrix GeneChip Human Arrays. Significantly differentially expressed transcripts between amniocytes and AFS were obtained by using Welch’s t-test. Unsupervised hierarchical clustering was used to visualize overall expression characteristics and differences in transcripts between AFS and amniocytes. The biological functions of selected genes were analyzed using various online Gene Ontology databases.

**Results:**

A total of 3,072 and 15,633 transcripts were detected in the second-trimester AFS and amniocytes, respectively. Hierarchical clustering revealed differential transcript expression between AFS and amniocytes. We found 353 genes that were specifically enriched in the AFS only, and tissue expression analysis showed enrichment of brain-specific genes in the AFS. Biological pathway analysis revealed that AFS-specific transcripts were mainly involved in embryonic development, cardiovascular development, and cellular morphology pathways.

**Conclusion:**

This study demonstrated differential tissue-specific gene expression profiles and biological pathways between AFS and amniocytes. The results suggested that AFS is the preferred RNA source to investigate potential biomarkers of fetal neurodevelopment.

## Background

Amniotic fluid is a dynamic solution that performs multiple functions for the developing fetus at different ages. During the second trimester, the amniotic fluid composition is similar to that of fetal plasma with rapid bi-directional diffusion via non-keratinized fetal skin between the fetus and the amniotic fluid [1]. Amniotic fluid is composed of cells, termed “amniocytes” and acellular fluid. Amniotic fluid supernatant (AFS) is the fraction of amniotic fluid after centrifugation. Amniocytes are derived from all three germ layers of the embryo, ranging from unspecified progenitors to mature differentiated cells. AFS contains suspended fetal transcripts including cell-free RNA and RNA released from amniocytes. The kinetics and origin of cell-free fetal RNA in the AFS have not been fully unraveled yet. Amniotic fluid mRNA can be associated with membrane-derived vesicles, which greatly enhance the mRNA stability and are released from healthy cells in exosomes or virtosomes. Additionally, cell-free RNA can be a product of either apoptosis or necrosis [2,3]. RNA of AFS consists of cell-free fetal RNA from fetal circulation and RNA particles from amniocytes. Cell-free mRNA can act as a messenger between cells and modify the biology of the recipient cells through changes in various cellular responses such as an immune response [3]. It is assumed that cell-free fetal RNA particles in amniotic fluid include cell-free RNA in fetal circulation, and that they play an important role in fetal development in health and disease.

Since the detection of cell-free fetal nucleic acids in maternal serum [4], the use of transcriptomes for prenatal diagnosis has rapidly developed. Cell-free fetal DNA represents a subfraction of 6–10% of total cell-free DNA in first- and second-trimester pregnancies and rises up to 10–20% in third-trimester pregnancies [5,6]. Cell-free fetal DNA is 100‒200-fold more abundant in amniotic fluid than in maternal plasma [7]. Amniotic fluid is an excellent source of material for research, as large quantities of supernatant can be obtained and there is no maternal nucleic acid contamination. In addition, amniotic fluid cell-free nucleic acids are more likely to originate from the fetus itself, in contrast to circulating cell-free fetal nucleic acids, which are predominantly of trophoblast origin [8]. Genomic analysis of cell-free fetal RNA from amniotic fluid can offer important real-time information on fetal physiology, development, and potential disease status in ongoing pregnancies. Therefore, it is important for studying human development and for antenatal diagnosis, and can provide clues for new biomarkers and therapeutic targets. In recent transcriptome studies of cell-free fetal RNA in AFS [9–12], diverse tissue-specific transcripts have been investigated, and it was found that neurodevelopment-related genes are especially abundant in mid-trimester AFS. All of these studies have used AFS samples, and no study has compared the differences in gene expression between AFS and amniocytes until date, except one previous [13] and the current study.

In this study, we investigated the tissue-specific expression patterns and their biological relevance in second-trimester amniotic fluid cell-free fetal RNA by comparing the transcriptomes of amniocytes and AFS. Specifically, we aimed to provide more information on the specific biological value of AFS cell-free RNA, which excludes the effect of amniocyte cellular RNA. We hypothesized that AFS is a more diverse source of fetal RNA and a more accurate biological marker for providing real-time information on fetal developmental physiology than RNA from amniocytes.

## Materials and Methods

### 2.1 Subjects

The ten pregnant women enrolled in this study were recruited from the department of Obstetrics and Gynecology, CHA Gangnam Medical Center, CHA Medical University (Seoul, Korea). This study was approved by the Ethical Review Board for Human Genome Studies at CHA Gangnam Medical Center, College of Medicine CHA University. We obtained written informed consent from all pregnant women to participate in the study. Karyotype results confirmed that the amniotic fluid samples were from 8 normal females and 2 normal males. The gestational age at amniocentesis ranged from 16 4/7 to 20 2/7 weeks. There were no pregnancy complications, and all babies had a normal postnatal outcome. Clinical information on the participants is presented in Table 1.

**Table 1:** Clinical information of the participants in this study.

No.	Karyotype	Age (yrs)	GA (wks+days)	Indication of amniocentesis
CHA-01	46,XY	38	17+3	advanced maternal age
CHA-02	46,XX	38	20+2	screening positive
CHA-03	46,XX	27	17+0	screening positive
CHA-04	46,XY	31	17+2	screening positive
CHA-07	46,XX	35	16+4	screening positive
CHA-33	46,XX	32	16+5	single umbilical artery
CHA-44	46,XX	36	17+3	screening positive
CHA-52	46,XX	40	18+6	advanced maternal age
CHA-80	46,XX	31	17+6	screening positive
CHA-85	46,XX	34	17+2	maternal anxiety (prev. Turner synd. pregnancy)

### 2.2 RNA extraction

Ten second-trimester amniotic fluid samples were centrifuged at 350 × *g* for 10 min at room temperature to separate amniocytes, and the amniocytes were cultured for one passage. The supernatants and amniocytes were stored at -70°C until use. Total RNA was extracted from the amniocytes by using the QIAamp RNeasy Mini Kit (Qiagen, Hilden, Germany). Total RNA was extracted from 10–20 ml of AFS by using the QIAamp Circulating Nucleic Acid Kit with carrier RNA (Qiagen) with an on-column DNase digestion step to remove genomic DNA according to the manufacturer’s instructions. The RNA was purified with the RNeasy MinElute Cleanup Kit (Qiagen). The RNA quality was analyzed using the Agilent 2100 Bioanalyzer and the RNA concentration in each sample was measured using a NanoDrop ND-1000 spectrophotometer (NanoDrop Technologies, Wilmington, DE, USA). The RNA samples with an A260:A280 ratio greater than 1.8 were stored at -70°C for further analysis.

### 2.3 Microarray

Gene expression was analyzed with the GeneChip PrimeView Human Gene Expression Array (Affymetrix, Santa Clara, CA, USA), which contains over 530,000 probes covering more than 36,000 transcripts and variants, which represent more than 20,000 genes mapped through RefSeq or via UniGene annotation. Biotinylated aRNA was prepared according to the standard Affymetrix protocol (Expression Analysis Technical Manual, 2001) from 100 ng total RNA using the GeneChip 3’ IVT Express Kit (Affymetrix). Following fragmentation, 12 μg of aRNA was hybridized to the GeneChip array for 16 h at 45°C. The GeneChips were washed and stained in the Affymetrix Fluidics Station 450, and scanned using the Affymetrix GeneChip Scanner 3000 7G. The data were analyzed with Robust Multi-array Analysis (RMA) using Affymetrix default analysis settings and global scaling as normalization method. The trimmed mean target intensity of each array was arbitrarily set to 100. The normalized and log-transformed intensity values were analyzed using GeneSpring 12.5 (Agilent Technologies, CA, USA).

### 2.4 Data analysis

The data were imported into the GeneSpring GX 7.3 software (Agilent Technologies) for filtering and basic statistical analysis. Signals were considered “detected” when their value was larger than the median value of control probe signal. Hierarchical clustering analyses were used to visualize overall expression characteristics of all samples. We compared gene expression in paired AFS and amniocyte samples. We identified genes that were differentially expressed in AFS, amniocytes, and both samples by using ANOVA followed by Benjamini-Hochberg multiple testing corrections. Genes were considered significantly differentially expressed in both AFS and amniocytes if they had a fold-change of at least 2 and a Benjamini-Hochberg adjusted *P*-value < 0.05.

Biological function pathway analysis was done using the web-based Ingenuity Pathway Analysis (IPA) software system, which identifies the pathways associated with a list of uploaded genes. In this analysis, we uploaded the three lists of differentially expressed genes as described above. A threshold of *P* < 0.05 was set to identify active functions. A *P*-value was calculated by Fisher's exact test to determine the significance of the association of the dataset and the specific pathway.

Additionally, the online Database for Annotation, Visualization and Integrated Discovery (DAVID) toolkit 6.7, an ontology-based webtool (http://david.abcc.ncifcrf.gov/), was utilized to statistically evaluate groups of genes identified in previous publications and other public resources for tissue-specific analysis. Similar to IPA, we uploaded the gene lists using the official gene symbols to identify enriched gene ontologies, which were selected by primary filtering.

## Results

We obtained 10 amniotic fluid samples from women in the second-trimester pregnancy. The median gestational age at amniocentesis was 17 weeks (range 16‒20 weeks). The mean maternal age was 34 years. The amniotic fluid samples were from 2 male and 8 female fetuses. The indication for amniocentesis was advanced maternal age or a positive serum screening test result (Table 1).

**Fig 1:**
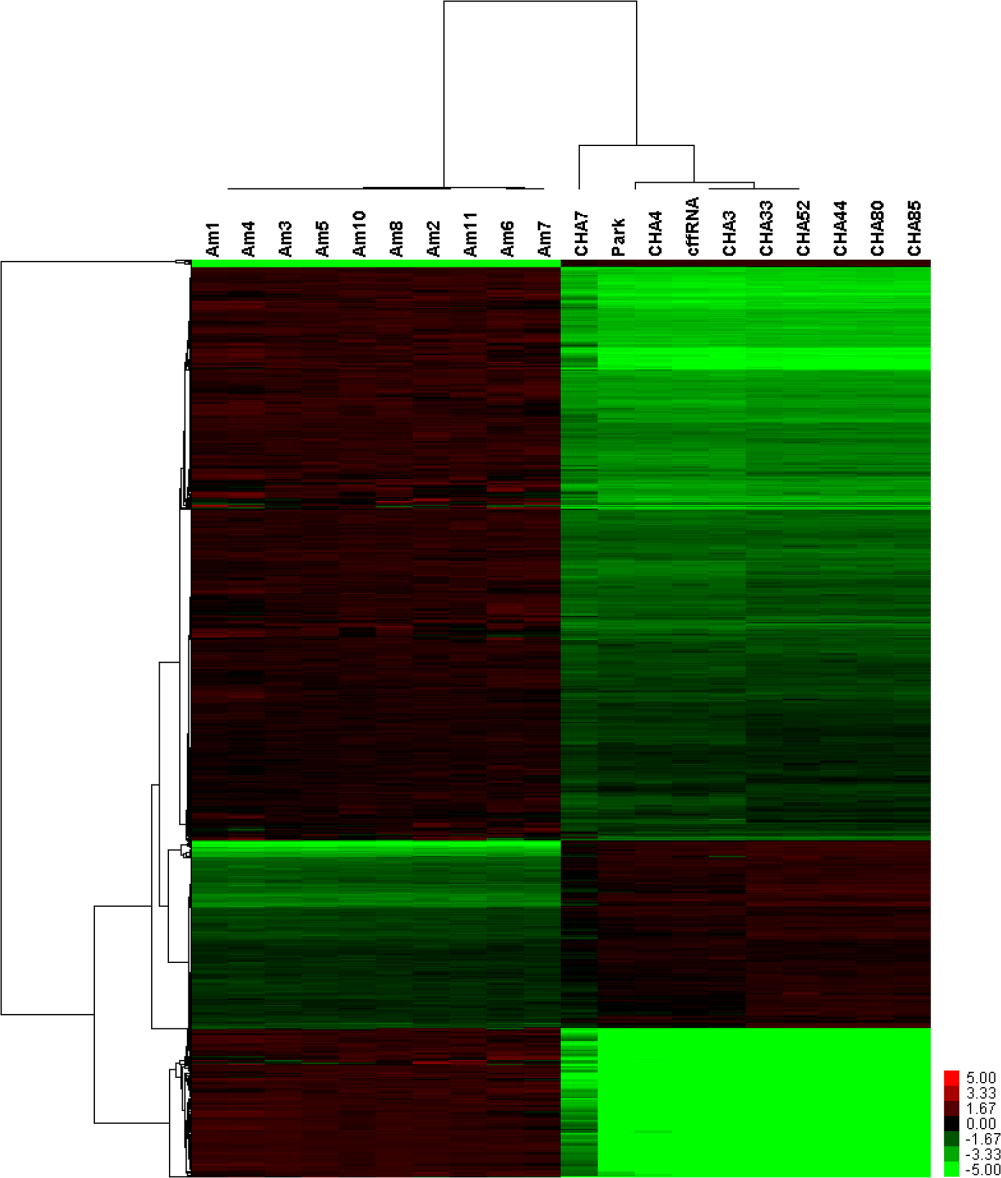
Unsupervised hierarchical clustering analysis for genes differentially expressed between amniocytes and amniotic fluid expression profiles.

**Fig 2:**
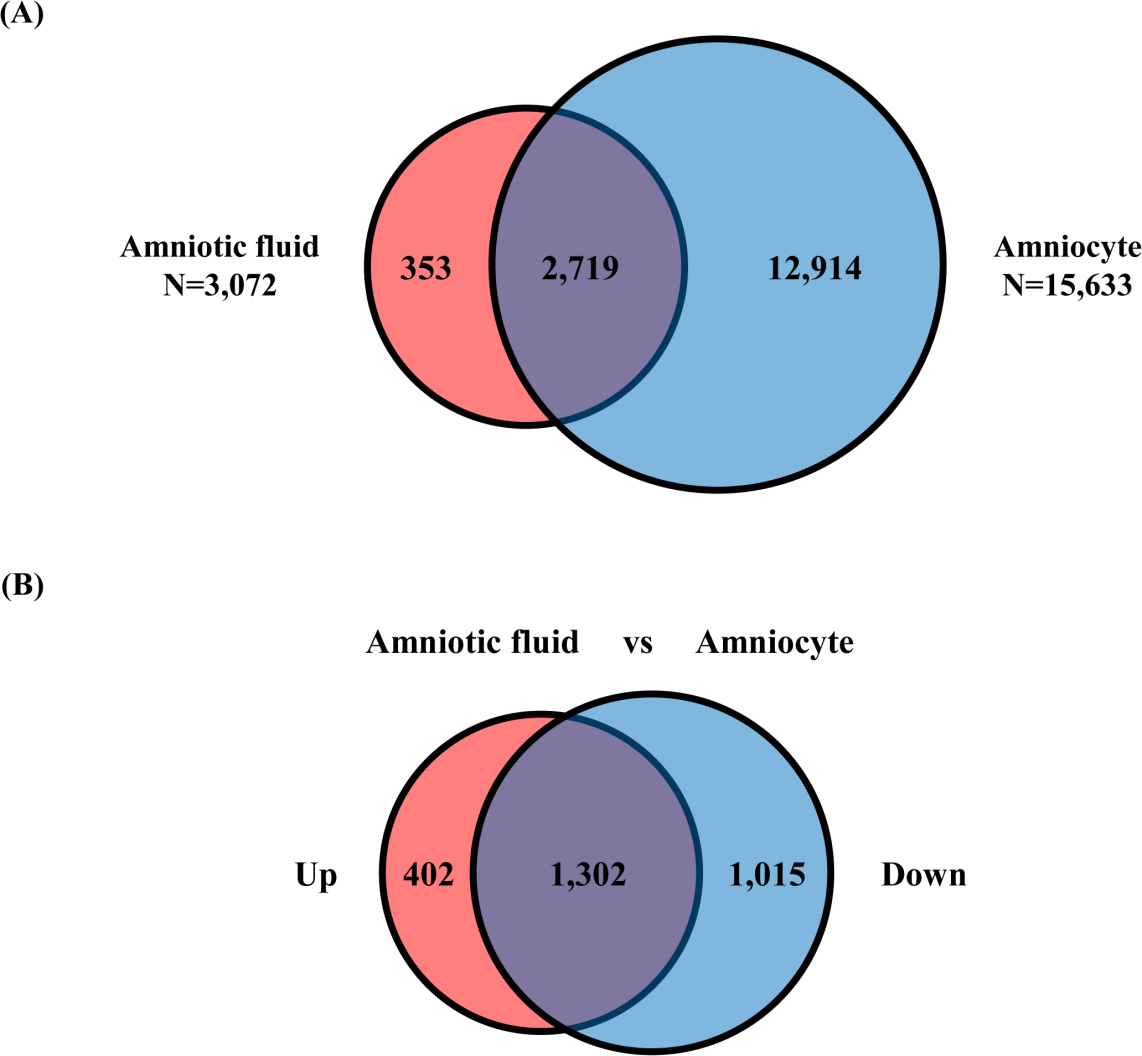
A. Venn diagram of the numbers of genes detected in the AFS and amniocytes by comparative transcriptome analysis. B. Venn diagram showing the numbers of up- and down-regulated genes that overlapped between the AFS and amniocytes.

The genome-wide expression profiles of amniocyte and AFS RNA were compared using PrimeView Human Gene Expression Arrays. Unsupervised hierarchical clustering showed that the AFS RNA profile was distinctly different from that of amniocytes (Fig 1). We found that 3,072 genes were expressed in AFS and 15,633 genes were expressed in amniocytes (S1 File). Of these, 353 genes were expressed uniquely in AFS, while 12,914 genes were expressed in amniocytes alone and 2,719 genes were expressed in both AFS and amniocytes (Fig 2A). Among the 2,719 genes expressed in both, 402 genes were up-regulated and 1,015 genes were down-regulated in AFS compared to amniocytes (Fig 2B). To identify whether the three different groups of detected genes showed tissue-specific expression, we performed DAVID analysis. Among the 353 genes uniquely expressed in the AFS, we identified 150 tissue-specific genes associated with the brain, spleen, blood, heart, saliva, and testis (Table 2) with most genes being expressed in the brain (119 genes) and spleen (11 genes). The amniocytes were mainly enriched for transcripts from the placenta, lung, epithelium, uterus, skin, and liver (Table 3). Among the 2,719 genes detected in both AFS and amniocytes, the transcripts mainly originated from the brain, placenta, lung, and uterus (Table 4). Detailed gene lists are supplied as supplementary files (S2 File, S3 File and S4 File).

**Table 2:** Tissue-specific genes uniquely expressed in the AFS.

Organ	*p*-value	BH value	No. of genes
Expressed exclusively in testis	0.008	0.684	5
Heart	0.018	0.833	4
Saliva	0.021	0.752	3
Blood	0.046	0.900	6
Brain	0.047	0.843	119
Spleen	0.053	0.827	11
Primary B-Cells	0.095	0.937	2

**Table 3:** Tissue-specific genes uniquely expressed in the amniocytes.

Organ	*p*-value	BH value	No. of genes
Umbilical cord blood	3.22E-29	1.19E-26	21
Skin	2.96E-28	5.50E-26	189
Cajal-Retzius cell	1.40E-27	1.73E-25	10
Epithelium	2.37E-26	2.20E-24	265
Fetal brain cortex	8.60E-24	6.38E-22	7
Placenta	2.23E-20	1.38E-18	380
Muscle	1.27E-18	6.74E-17	59
Lung	2.06E-16	1.03E-14	272
B-cell lymphoma	1.99E-14	8.19E-13	3
Cervix carcinoma	2.15E-14	7.99E-13	14
Uterus	2.29E-14	7.71E-13	222
Platelet	9.42E-14	2.91E-12	18
Cervix	1.72E-11	4.92E-10	31
Urinary bladder	3.74E-09	9.92E-08	10
Liver	4.47E-09	1.11E-07	109
Eye	2.73E-08	6.32E-07	62
Pancreas	8.64E-08	1.88E-06	45
Kidney	1.22E-07	2.52E-06	92
Lymph	1.66E-07	3.23E-06	33
Ovarian carcinoma	2.03E-07	3.76E-06	2
Promyelocytic leukemia	2.22E-07	3.92E-06	2
Ovary	2.37E-07	4.00E-06	63
Bone marrow	2.44E-07	3.94E-06	37
Keratinocyte	6.58E-07	1.02E-05	5
Hepatoma	6.35E-06	9.43E-05	8
Colon adenocarcinoma	9.02E-06	1.29E-04	1
Adrenal gland	1.05E-05	1.44E-04	11
T-cell	3.27E-05	4.34E-04	6
Prostate	2.15E-04	0.0027429	35
Coronary artery	2.40E-04	0.002965	1
Dendritic cell	4.62E-04	0.0055176	4
Fibroblast	6.51E-04	0.0075272	3

**Table 4:** Tissue-specific genes expressed in both AFS and amniocytes.

Organ	*p-value*	BH value	No. of genes
Skin	3.82E-22	1.10E-19	38
Muscle	1.05E-18	1.51E-16	9
Epithelium	2.14E-15	2.02E-13	31
Uterus	3.98E-15	2.87E-13	41
Placenta	1.19E-14	6.82E-13	172
Lung	2.22E-14	1.06E-12	42
Cervix	7.15E-10	2.28E-08	2
Pancreas	6.58E-09	1.89E-07	12
Cervix carcinoma	9.53E-09	2.49E-07	1
Ovary	1.93E-08	4.62E-07	15
Eye	6.45E-08	1.32E-06	13
Platelet	8.79E-08	1.68E-06	1
Liver	4.55E-07	7.25E-06	29
Brain	9.08E-07	1.37E-05	388
Kidney	3.08E-06	4.42E-05	18
Lymph	9.36E-06	1.28E-04	4
Ovarian carcinoma	1.04E-05	1.36E-04	2
Colon	5.21E-05	6.23E-04	38
B-cell	8.02E-04	0.008487	2

Pathway enrichment analysis by IPA to examine the roles of differentially expressed genes (DEGs) in the AFS samples revealed 61 functional categories. Table 5 presents the genes that are more than 10-fold up-regulated and with *P*-value < 0.01 including the categories that are related to general organ systems and functionality. Embryonic development, cardiovascular development, and cell morphology pathways were enriched biological functions in genes uniquely expressed in the AFS. The genes uniquely expressed in amniocytes were mainly enriched in infectious disease, cell death and survival, and protein synthesis pathways (Table 6). Functional analyses of DEGs in both AFS and amniocytes showed that particular genes were significantly differentially regulated in AFS compared to amniocytes (Table 7). SPON2, known to be involved in the clearance of influenza viruses, is up-regulated more than 30-fold in AFS compared to amniocytes. Other up-regulated genes included LRFN1, NFIC, ATG9B, TBC1D16, and GPRIN1. Genes that were down-regulated in AFS comprised RPL34, PSMA6, and UBC, which are involved in protein synthesis and degradation.

**Table 5:** Top biological functions identified in unique genes of AFS by Ingenuity Pathway Analysis (IPA).

Category	*p* value range	No. of genes	Associated function
Behavior	9.90E-05	5	Mechanical allodynia behavior
Connective Tissue Disorders, Skeletal and Muscular	1.40E-03	2	Synostosis of cranium
Embryonic Development, Organismal Development	2.23E-04 ~ 1.40E-03	30	Development of body trunk, neuroectoderm, rhombomere 1
Cardiovascular System Development and Function	3.48E-04 ~ 9.12E-03	32	Activation and contraction of heart, cardiogenesis, morphogenesis, growth of blood vessel
Cell Morphology, Cellular Compromise, Inflammatory	4.28E-04 ~ 2.39E-02	14	Swelling of cisternae, breakage of cellular membrane, disorganization of cells etc.
Organismal Injury and Abnormalities	6.57E-04 ~ 8.69E-03	20	Strength of skeletal muscle, fibrosis of tissue, damage of lung
Gene Expression	8.48E-04 ~ 4.29E-03	6	Binding of octamer element and synthetic promoter
Skeletal and Muscular System Development and Function	8.48E-04 ~ 1.20E-02	5	Strength of skeletal muscle, deposition of cartilage matrix, force generation of muscle and myotube
Embryonic Development, Nervous System	1.20E-03 ~ 6.00E-03	7	Formation of mesencephalon and cerebellar cortex
Cell Death and Survival	1.21E-03 ~ 2.39E-02	33	Activation-induced cell death, loss of neurons, etc.

**Table 6:** Top biological functions identified in unique genes of amniocytes by Ingenuity pathway analysis (IPA).

Category	*p* value range	No. of genes	Associated function
Infectious Disease	8.46E-17 ~ 6.82E-08	416	Viral Infection, infection of cells, replication of Influenza A virus
RNA Post-Transcriptional Skeletal and Muscular	1.65E-16	105	Processing of RNA
Post-Translational Modification	3.38E-14 ~ 2.25E-09	135	Ubiquitination, folding of protein, conformational modification of protein
Cell Death and Survival	4.87E-14 ~6.79E-08	783	Apoptosis, necrosis, cell viability of tumor cell lines
Protein Synthesis	5.34E-13 ~ 5.24E-08	274	Translation of protein, initiation of translation of protein, metabolism of protein, expression of protein
Cellular Growth and Proliferation	1.52E-10	782	Proliferation of cells
Cancer	6.58E-12 ~ 4.62E-09	424	Mammary tumor, colon cancer, breast or ovarian cancer
Dermatological Diseases	1.11E-09	50	Chronic psoriasis
Neurological Disease	1.29E-09 ~ 7.47E-09	217	Disorder of basal ganglia, Movement Disorders
DNA Replication, Recombination, and Repair, Energy Production, Nucleic Acid Metabolism, Small Molecule Biochemistry	1.27E-08 ~ 4.19E-08	129	Catabolism of nucleoside triphosphate, repair of DNA

**Table 7:** Particular genes significantly differentially regulated in AFS compared to amniocytes.

Up-regulated genes			
Bio-function	Gene name	Description	FC
Cell Death and Survival	CTBP1	C-terminal binding protein 1	2.4
	NFIC	nuclear factor I/C (CCAAT-binding transcription factor)	4
	SMURF1	SMAD specific E3 ubiquitin protein ligase 1	2.8
	LRFN1	leucine rich repeat and fibronectin type III domain containing 1	6.3
	MED29	mediator complex subunit 29	2.5
Cellular Function and Maintenance	ATG9B	ATG9 autophagy related 9 homolog B	3.6
	HERC1	hect (homologous to the E6-AP (UBE3A) carboxyl terminus) domain	2.1
	SYNPO2	synaptopodin 2	2.4
	TBC1D16	TBC1 domain family, member 16	3.2
	TBC1D17	TBC1 domain family, member 17	2.3
	GPRIN1	G protein regulated inducer of neurite outgrowth 1	3.6
Antimicrobial Response	HLA-A	major histocompatibility complex, class I, A	2.9
	SPON2	spondin 2, extracellular matrix protein	33.3
Cardiovascular System	MB	myoglobin	2.3
	THRA	thyroid hormone receptor, alpha (erythroblastic leukemia viral (v-erb-a) oncogene homolog, avian)	2.7
**Down-regulated genes**			
**Bio-function**	**Gene name**	**Description**	**FC**
Cell Death and Survival	AP1G1	adaptor-related protein complex 1, gamma 1 subunit	6.5
	MAFG	v-maf musculoaponeurotic fibrosarcoma oncogene homolog G	22.6
	TEAD2	TEA domain family member 2	14.1
Cancer	IRX5	iroquois homeobox 5	2.2
	PRICKLE1	prickle homolog 1 (Drosophila)	18.7
	RANBP3	RAN binding protein 3	14.3
	RPL34	ribosomal protein L34	97.4
	BAZ1B	bromodomain adjacent to zinc finger domain, 1B	25.9
	CHD4	chromodomain helicase DNA binding protein 4	8.2
	PSMA6	proteasome (prosome, macropain) subunit, alpha type, 6	189.8
	UBAC1	UBA domain containing 1	5
Protein Synthesis	APEH	N-acylaminoacyl-peptide hydrolase	16.8
	BANP	BTG3 associated nuclear protein	3
	D2HGDH	D-2-hydroxyglutarate dehydrogenase	3.3
	PMPCA	peptidase (mitochondrial processing) alph	20.9
	PPP2R3A	protein phosphatase 2, regulatory subunit B'', alpha	15.1
	SGSM3	small G protein signaling modulator 3	13.3
	UBC	ubiquitin C	67.89
	USP19	ubiquitin specific peptidase 19	2.8
Cellular Movement	GDI1	GDP dissociation inhibitor 1	21.5
	SNX27	sorting nexin family member 27	8.7

FC: Fold change, indicates up-regulation in AFS RNA relative to amniocytes, respectively. This table lists genes that showed FC > 2.0.

## Discussion

In this study, we investigated tissue-specific expression patterns enriched in second- trimester amniotic fluid cell-free fetal RNA and compared them with those in second-trimester amniocytes by using transcriptome analysis. To our knowledge, this is the first published study comparing global gene expression of amniocytes and AFS. Our results demonstrated different RNA expression patterns in tissue-specific genes and biological pathways between both sample types. Although the possible effects of the *in vivo* half-life of fetal transcripts in AFS should be considered, the AFS contains less genetic information than the amniocytes. We detected 353 genes that were unique to the supernatant. These included genes specific to the brain, spleen, blood, testis, heart, and saliva. The brain-specific transcripts were highly represented, with 119 genes. The 12,914 genes that were uniquely expressed in the amniocytes were specific to more diverse organs and mainly originated from the umbilical cord blood, skin, placenta, lung, epithelium, uterus, and liver. Additionally, in cellular RNA of amniocytes, tissue-specific genes of putatively non-fetal origin (umbilical cord, placenta, and uterus) were abundant. Genes that were common between AFS and amniocytes were specific to the brain, skin, uterus, placenta, and lung. Particularly brain-specific genes were enriched in the AFS alone and in the genes common in both amniocytes and AFS. It must be that brain development is main fetal developmental process during second trimester pregnancy. Our results were consistent with previous findings that brain-specific genes are important in second-trimester fetal development [9–12]. It is assumed that they play an important role in fetal brain development and can provide new biomarkers for monitoring of brain development. Genes specific to testis, spleen, heart, saliva, and primary B-cells are present only in AFS. Future studies should include a more in-depth evaluation of the roles of these genes in fetal development.

Functional analysis of the DEGs showed that the highly expressed genes in second trimester AFS and amniocytes were involved in different pathways; while the AFS was enriched for genes involved in embryonic and cardiovascular development, and cell morphology, the pathways regulating infectious disease, cell death and survival, and protein synthesis were represented only in the amniocytes. Among the genes expressed in both AFS and amniocytes, those involved in cell death and survival and antimicrobial response were up-regulated, while genes of cancer and protein synthesis pathway were down-regulated. As the AFS was enriched for genes implicated in cardiovascular and embryonic development, and cell morphology, AFS might be the preferred sample to study these processes in developing fetus. In cultured amniocytes, the cellular RNA is likely affected by the culture environment. There are up-regulated genes related to cell-dogma, as transcription, translation, and replication, and response to growth stimulation, as apoptosis, necrosis, and cell proliferation. Notably, the embryonic development pathway was represented only in the AFS, corroborating that AFS RNA—without any effect of amniocyte RNA—is the preferred source to search for potential biomarkers of fetal development.

Several studies have shown that AFS transcriptome analysis can provide information on the development of a number of different organs in living human fetuses. Previous AFS transcriptome studies on fetuses with twin-to-twin transfusion syndrome [14], Down syndrome [9], trisomy 18 [10, 11], and Turner syndrome [15], revealed that several genes and biological pathways were associated with the fetal developmental pathophysiology. AFS transcripts could play an important role in systemic cell-to-cell signaling and modulation of the fetal physiology.

In this study, we provided a list of genes that are highly expressed in amniocytes and AFS in mid-trimester. The transcriptome analysis of the AFS suggested that fetal brain-specific RNA appears to be a major source of amniotic fluid RNA, without the effect of the amniocytes. Therefore, AFS RNA might be useful to uncover the mechanism of development of the central nervous system (CNS) in fetuses and to find biomarkers for and the pathogenic mechanism of CNS disorders. Future studies could compare the transcriptomes of fetuses with CNS anomalies, unknown intrauterine growth restriction, unknown intrauterine fetal death, and unknown cerebral palsy, with those of normal fetuses based on AFS samples that are devoid of uncultured amniocyte effects. We expect that this study will provide a foundation for future studies on differential gene expression of amniotic fluid in abnormal fetal development and on the influences of amniotic fluid and amniocytes on normal fetal development in mid-trimester pregnancies.

This study had several limitations. Firstly, the sample size (10 samples) was small. Secondly, there were differences in fetal gender (female sex dominance), gestational age, and maternal BMI in the samples, and fetal gene expression patterns in AFS vary according to these factors [14, 16, 17]. Thirdly, the stability of RNA from AFS and amniocytes differs; cell-free RNA is relatively unstable compared to cellular RNA, suggesting that nucleic acid degradation might have affected the results. Finally, culture of amniocytes could change the RNA expression in the cells. Therefore, this study provided preliminary data, and future studies with larger sample sizes, uncultured amniocytes, and controlled confounding factors will be needed to analyze the AFS transcriptome in more detail and to reveal markers for fetal developmental physiology.

In conclusion, we found distinct differences in gene expression, tissue-specific genes, and biological pathway in AFS and amniocytes. The transcriptome of AFS alone was enriched for fetal brain-specific transcripts in comparison to the amniocyte transcriptome. We showed that AFS is indeed a valuable source of RNA and a might be a source of important biological markers providing real-time information on fetal development physiology. Future transcriptome studies of cell-free RNA in amniotic fluid from live pregnancies with developmental disorders can permit advanced understanding of the early etiology of disease and may suggest innovative approaches to treatment.

## Supporting Information

S1 File Lists of genes differentially expressed in AFS, amniocytes, and both samples. (XLSX)

S2 File Detailed lists of tissue-specific genes expressed in AFS. (XLSX)

S3 File Detailed lists of tissue-specific genes expressed in amniocytes. (XLSX)

S4 File Detailed lists of tissue-specific genes expressed in both AFS and amniocytes. (XLSX)
